# Students’ attitude and academic achievement in a flipped classroom

**DOI:** 10.1016/j.heliyon.2022.e08792

**Published:** 2022-01-22

**Authors:** Cecilia Obi Nja, Richard Ekonesi Orim, Hope Amba Neji, John Okpa Ukwetang, Uduak Edet Uwe, Mary Anari Ideba

**Affiliations:** University of Calabar, P.MB 1115, Calabar, Nigeria

**Keywords:** Flipped classroom, Students' achievement, Teachers and attitude

## Abstract

A quasi non-equivalent, non-randomized factorial design, examined Chemistry students' attitudes towards chemistry and academic achievement in second year Bachelor of Education students taught with the flipped classroom strategy. In the study of 100 students, a pre-attitude test of 30-items questionnaire was apportioned to ascertained students' attitude towards chemistry. Pre-test was also apportioned to the control and experimental group. This was followed by teaching using the flipped classroom strategy for experimental group and the control group was taught using the traditional approach. Thereafter, students in the experimental group wrote a post attitude test. A post-test was administered to both experimental and control group. The result of the post-attitude showed a significantly higher mean when compared to the pre attitude score. The result of the analysis using sample t-test showed students having a positive attitude towards chemistry, when taught using flipped classroom. The study also sought to find out the academic achievement of students taught with and without the flipped classroom strategy. Findings from the investigation of academic achievement revealed that students’ academic achievement was significantly higher than those in the conventional group. The implications of these findings are that the flipped classroom strategy could improve attitude of students towards chemistry, thereby enhancing their academic achievement. This is so as students can play the lesson videos again and again at the comfort of their homes for mastery of concept in their lesson. The result of this study is especially relevant to the learning and teaching of chemistry in secondary and tertiary institutions in developing nations, Nigeria inclusive. This is so as the teacher per student ratio is high such that the teacher cannot carry every student along in the class within the short period provided.

## 1 Introduction

Standard teaching which involves the inculcation of good knowledge with the purpose to enhance quality service delivery so as to meet man's requirement for food, health care facilities, and a host of other materials to advance the quality of life that humans live is termed chemistry education (Khanam, 2018). Chemistry is that part of Science that is involved with the understanding of matter. It also includes the study of the composition, properties, structures, and metamorphosis that occur in the universe (Ojokuku, 2012). Chemistry is an unrivaled essential part of science, applied science, and industry. It also constitutes the basis for life sciences (Mahdi, 2014).

Chemistry is a fundamental subject needed to combine with other lifelike science subjects such as physics, and biology to guarantee learners admission into higher institutions. Joint Admission and Matriculation Board (JAMB) (2020/2021UME) Brochure spelled out the guidelines for admission into first degree courses in Nigerian universities clearly stated that the mandatory subjects for all courses in medical/pharmaceutical and health science, five '0′ level credit passes in English Language, mathematics, physics, chemistry, and biology are necessary. For all courses in sciences, five '0′ level credits including English Language, chemistry, mathematics, physics, and biology. In engineering/environmental technology, education science, computer and agricultural science, chemistry is required (Jamb, 2021). The importance of this subject for students' future career aspirations in that academic and professional disciplines cannot be overemphasized. Review Reports Unfortunately, the performance and attitude of students in this important subject have not been encouraging (Abulude, 2016 and Nja et al., 2017).

In as much as chemistry is needed for a technologically driven society, chemistry students in Nigeria perform below expectation. Most of the chemistry candidates exhibit a lack of accuracy in chemical formulas and to balance chemical equations in types of reactions. This is found in Redox reactions reversible reactions and all types of balanced equations (Upahi and Olorundare, 2012). Other topics in chemistry that students find difficulties in answering questions include; Redox reactions, Electrode potential and electrochemical cells, Laws of electrolysis, the nomenclature of organic compounds, alkynes, chemical equation, particulate nature of matter, and chemical combination (Gongden et al, 2011, Jimoh, 2020).

Marcela and Mala (2016), the study discovered that learner's attitudes toward school were a determinant factor that predicts their academic achievement. The implication of the study, therefore, is that a positive attitude gives rise to positive results while a negative attitude turns out a negative result. Attitude refers to the predisposition to categorize objects and events, to react to them with assessment consistency. Attitude consists of one's information, reverence, emotions, incitement, and self-esteem designing an individual's outlook on a certain discipline. Students exhibit a certain attitude towards chemistry by reacting according to their understanding rather than to its actual state. In a study conducted by Hacieminoglu (2015) on Students' Attitude toward Science, the findings showed that students who had a more positive attitude towards science had higher achievement scores.

Attitude can be said to be the emotional and mental entities that propel an individual to take any action towards an object or subject (Perloff, 2016). Attitude is also the way the mind is disposed of, feels, or conditioned toward an individual or object (Khan and Ali, 2012). Attitude in chemistry is one's disposition toward the study of chemistry which can be a positive or negative feeling about chemistry. How one looks at chemistry be it difficult or simple (Salta and Tzougraki, 2004).

Facts about learners' attitudes with regards to chemistry scholarship are enveloped in the levels, how easy or hard to do, the intangible nature of some concepts, and the instructional technique and methods employed in class during teaching (Chua and Karpudewan, 2017). An expression such as ''chemistry is boring'' or ''The time for chemistry class is too long" or ''chemistry is very abstract'' or I feel sick when it is time for chemistry class" are some of the ways about the proofs that scholars view the learning of chemistry across -the -board. This aspect of affective domain expressed in words tend to show how the direction of the students' attitude toward chemistry; positive or negative towards chemistry as a subject and the related careers. Those with a positive attitude are motivated to work hard and this is reflected in the good mark scored in the examination.

Learners' attitude regarding the scholarship of science is an essential part of the aim of science instruction. The inculcation of scientific attitude during the learning of science and students' attitudes toward science is very crucial in a science class (Can and Boz, 2012). The three key factors according to Hofstein and Mamlok-Naaman (2011) which may influence therefore increase students' attitudes are; methodology of presentation of the content, resources and teaching aids used as well as gender bias. The study carried out by Juma and Ahmad (2012) revealed an expressive affinity between learners' attitudes concerning Information Technology and scholars' intellectual achievement revealed that the intellectual achievement and attitudes are interwoven. This research suggested that it is important to improve attitudes to increase student's academic achievement.

Burcin & Leman (2010) study revealed that active learning brought about a significantly better gain of scientific conceptions. Students who were taught in an active classroom setting developed more positive attitudes toward chemistry lessons when compared with those taught with the traditional method. In a similar vein, the work of Mello and Less (2013), Momani et al. (2016), and Karma and Tshering (2020) showed that an active learning approach enhanced the learning achievement of students in science. It also revealed that active learning promoted student motivation which resulted in improved academic performance. This is so as students who were involved in active learning environments had a more appreciated view of learning experiences compared to passive learning environment learners. Niemi et al. (2016), work revealed that active learning is necessary for the classroom as it makes learning permanent.

Among other aims of chemistry, learning is to nurture students' positive attitudes towards chemistry Hofstein and Mamlok-Naaman (2011), Kumari and Saraladevi (2014) and Kemendikbud (2017). Outcomes of attitude in teaching and learning are as relevant as the cognitive domain. It is disheartening to note that, majority of conventional teaching and learning of science teachers concentrate on the cognitive field. Paramitha's (2017), the study revealed that Students' interest and attitude in learning are the predominant factors in the mastery of any subject matter.

The use of innovative technologies in educational practices to enhance academic performance in higher education institutions has become a persistent concern (Deng and Tavares, 2013). There is a paradigm shift in students' learning in the twenty-first century from teacher-centered to learnercentered. Learners do not sit passively in class while the teacher spits out knowledge to them but learners are actively involved in the teaching and learning process. It is no longer believed that learners are blank slates that sit in front of the teacher waiting for information to be written on their blank minds. This, therefore, implied that ideologies, methods, practices, and systems hitherto used in teaching need to be overhauled. Educational institutions are required to adjust their system and learning environment to cater to the paradigm shift in the educational system (Paje, 2013).

Investigation into causes of low academic achievement in such topics revealed that; learning styles, none availability and utilization of instructional materials, use of foreign examples that students are not familiar with, large class size, gender, lack of textbooks, unqualified teachers, teaching methods, school location, students attitude toward studying science, poor study habits, lack of interest in school and test phobia affect achievement (Neji et, al., 2013, Idris 2015, Ojukwu, 2016 & Idiege et al., 2017).

There are diverse ways in which students learn. It can be through auditory, taking of notes and visualizing, (Naimie et al., 2010). Computer laboratories are established by many schools to enhance students' concentration in terms of what they hear, vocalize, and communication skills (Yağcıoğlu, 2008). The creation of multi-media (audio-video) is to serve as supplementary activities for practice, procedure, control, inestimable time, and heterogeneous learning. Acquisition of innovative skills can be developed through computer-assisted instruction using suitable approaches and pedagogical techniques (Seljan, et, al., 2006).

Supplementary video related to this article can be found at https://doi.org/10.1016/j.heliyon.2022.e08792

The following are the supplementary data related to this article:

1. introduction.redox 1 1. introduction.redox

2. Balance_Redox_Reactions_ 2 2. Balance_Redox_Reactions_

In 2018, Galanek et al. conducted an annual study of undergraduate students' information technology acquisition and usage. It was discovered that Laptops were king, smartphones were queen, and tablets were going into extinction. The results showed that at least 99% of students possess a smartphone or tablet, and 30% of students have a laptop. ICT was seen as a critical tool for students' academic success. This was so as three-quarters of students said their smartphones were of average importance. In another study that was conducted by Ejechi (2016) in Nigeria University, it was revealed that information on possession of cell phones, laptops, and the desktop was 100%, 30.9%, and 1.0% respectively. Due to the number of hours spent by students on technology, it is eminent to add learning content into technology to attract students to study. With the course content in the University increasing and the time to finish the course content decreasing, many students lagged. The effective usage of time outside the classroom becomes unavoidable. Technology can be of great gain to in-class and out-of-class time when implemented correctly.

Flipping the class which is a blend of technology and student active learning, was initiated as a result of the need to enhance learning in the classroom and outside the classroom (Saglam and Arslan, 2018). Lately, the concept of the flipped classroom has become a rampant method of instruction in schools. The flipped learning has many forms; one of which is blended learning. Garrison and Kanuka (2004), defined blended learning as the incorporation of traditional teaching activities with online learning activities.

A flipped classroom is the opposite of the traditional method of teaching in which, teaching is done in the class and students take-home assignments. Teaching in the flipped classroom entails that the teacher initiates video lessons or gets the video already prepared for use in the class. This video is given to the students to learn at home before its use in the classroom. The teacher must provide professional, additional service to the students. With the flipped class more time for active learning is created, there is an effective use of time in the classroom as the teacher answers questions that students brought to the classroom that they have issues with (Cabi, 2018; Hessler, 2016). In a flipped classroom, students' attention is enhanced as the class is participatory and there is a change from the normal sit and listen to individual learning (Jose and Jorge 2020; Birgili et al., 2021). Students' understanding of concepts becomes better in the flipped classroom (Hava 2021).

Proponents of the flipped classroom believe that it enhances the teacher-student relationship, enable deep learning utilizing effective classroom participation, enables a student to comprehend their learning technique and options, and bring about active involvement in learning (McLean et al., 2016). During teaching and learning in the flipped classroom arrangement, there was an increased learning exercise, improved self-regulative abilities, and explicit learning approaches. This could have been responsible for the efficient outcome of the flipped classroom thereby increasing learning outcomes (Lag and Sæ le, 2019). Newman et al., (2016) discovered that learning improved without respect to age, sex, and students' educational levels.

Studies done on the flipped classroom earlier indicated that students in the flipped classroom environment had higher academic achievement, had a positive attitude in learning. This was not unconnected to the fact that flipped classroom reduces stress, allows students to be responsible for their learning, and encourages collaborative learning (Strayer, 2012; Marlowe, 2012).

Flipping the classroom occurs when the teaching activities that were conventionally undertaken by the learner outside the class like, assignments and home works are shifted into the classroom, and that which was conventionally accomplished in class is achieved before coming to class (Lag and Sæ le, 2019). The rise in technological tools which include interactive videos, learner centers, and video ingathering programs has facilitated the extensive usage of flipped classrooms. Flipped study hall is among one of the educating and learning methodologies that support and advance teaching and learning with the assistance of mechanical gadgets, Once more, the subtleties of substance are conveyed to the learners outside the study hall period utilizing innovation apparatuses, for example, video. This recording can be made by the educator or sourced for on the web. These recordings clarify the idea the educator plans to convey to students (Tully, 2014; Johnston, 2017).

The teacher in the flipped classroom uses technological means to enhance flipped classrooms. Videos prepared by the teacher or source are presented to the students via YouTube for Educational purposes, TED Talk, Khan, or Academy (Koc, 2016). Flipped classroom approach takes cognizance of individual differences of students, this leads to improved students' academic achievement as boredom disappears and excitement and pleasurable learning increases. Students come into the classroom learning environment after they have reviewed learning materials and are ready to harness what was previously learned at home. The teacher commences the teaching process by checking students' degree of comprehension and reflects on what was previously studied at home; then he/she introduces the lesson. There are no home assignments in the flipped classroom as student's home assignments activities are carried out in the classroom.

Strohmyer (2016) asserted that applying flipped classroom strategy enables teachers to make good use of classroom period, enhances student's critical thinking and self-learning as well as building experiences and communication skills. This can lead to cooperation among students. Alshahry (2015) sees flipped classroom strategy as an approach that is capable of changing the duty of the instructor from one who spits out knowledge to a facilitator and guardian, and making students become researchers that get effectively involved in the teaching and learning processes, it will also help students to learn in line with their capacities as well as their uniqueness. Finally, it will bring about the excitement in the educational environment which will enhance desirable cognitive talent like rational creativity. Alzain's (2015) view of flipping the classroom is that it will bring about the utilization of modern technological solutions to tackle academic weaknesses. Alzain (2015) also added that flipped classrooms can lead to improve intellectual abilities of learners. When a flipped classroom is well articulated, it can stimulate and excite learners to be actively involved in the learning process instead of being docile receivers of information (Hunley, 2016).

In as much as the flipped classroom method of instruction is gaining ground in the western world, little or not much has been done in the University of Calabar on the use of the flipped classroom. This research paper, therefore, sought to find out University of Calabar Science Education students' attitudes and academic performance in a flipped classroom. This paper examined Science Education undergraduates’ students' who were in their second year. Their pre and post attitudes towards chemistry in a flipped classroom. The study also investigated students' academic performance in the flipped classroom.

## 2 The Nigeria context

In Nigeria, studies on the evaluation of predictors of undergraduates' university scholars' attitudes concerning chemistry are scanty. Most studies are on secondary school Chemistry. These studies enumerated the following: teaching methods, career interest, teacher attitude, age, cognitive styles of the student, influence of parents, social view of science, gender and social implicating of science (chemistry), and performance in chemistry. The flipped classroom was not investigated on its impact on undergraduate academic achievement and student attitude. This study, therefore, was framed to probe into an optional tutoring situation such as a flipped classroom. It was to find out its influence on scholars' attitudes and achievement in REDOX reaction. In a chemistry class, active participation and visualization of abstract concepts would be enhanced through the use of a flipped classroom (Estapa and Nadolny, 2015; Hsu et al., 2017). The merits of audio-visual aids include; learners' interest in learning concepts, visualization of presented chemical activities. Audio-visual movies used in a flipped classroom enable scholars to conceive unique intellectual figures of chemistry and enhanced long-lasting knowledge of the learned phenomenon. Student learning was facilitated as it empowered visualization in the teaching of mathematics, which aid continual learning (Zengin, 2017).

Still in Nigeria, even though the teaching of chemistry is supposed to be pragmatic, chemistry teaching had witnessed numerous hindrances. These are connected to the issues of lack of functional rooms to carry out practical chemistry lessons in both secondary schools and tertiary institutions of learning. A few of the hindrances included an absence of chemicals, students' population explosion, and few weeks for a semester and therefore time for practical not sufficient, and a dearth of concrete laboratory materials for maintaining learners' pragmatic experiences (Faour and Ayoubi, 2018 & Chao et al., 2015). The situation has also been worsened by Covid-19 that has reduced the contact time between teachers and students. The education sector in Nigeria receives the least budget allocation. Nigeria trails seriously behind the mandatory 26% benchmark of budgetary allocation for education, according to United Nation standard. This is so as education allocation in 2021 is 5.6%. This has led to schools being poorly funded and as such resources and facilities are scarce for experiments during teaching and learning in chemistry.

A quick check on the teaching and learning of chemistry shows that a positive attitude toward science is lacking, an attempt to handle this deficiency in school chemistry is therefore necessary. It calls for an announcement of a crisis that needs remediation and the focus should be on availability, fresh innovative techniques in the teaching and learning environment, this has not yielded any result because of scarce resources (Wallet, 2015). The prevalent view on Information and Communication Technology (ICT) is that it could bring about requisite reforms in the education system (Isaacs, 2012). As it is in Nigerian schools, accessing smartboards, computers, tablets, and the free-Wi-Fi connection is a mirage. For teachers to appropriately make use of ICT to teach without the smartboard, computers, and large lecture room the flipped classroom is necessary for the University of Calabar (Osborne et al., 2003). The desire to inquire how attitude influences learning is linked to the overwhelming relationship found in the previously reviewed literature (Meşe and Sevilen, 2021; Rana et al., 2015). Students value demonstrations, practical, hands-on experimentation. Such a classroom that is activity-laden results in students expressing a positive attitude towards science and mathematics (Ledic et al., 2012).

The research questions that directed the research work include: (1) what is the statistical meaningful variance in second-year BSc.Ed student pre and post-attitude towards learning instruction with the flipped classroom? (2) Is there a significant influence of attitudes on chemistry students' performance in chemistry? (3) How is the academic performance of second-year BSc.Ed students taught using flipped and non-flipped classroom?

### 2.1 Research design and methodology

A quasi non-equivalent, non-randomized factorial design. It is a modification of pretest posttest control group design with one treatment variable one moderator variables. The factorial 2 × 2 design is simply:

$$\begin{array}{l} \begin{array}{lllll} \text{Y} & {\text{O}}_{1} & {\text{x}}_{1} & 0_{2} & \text{E} \end{array}\\ \begin{array}{llll} {\text{O}}_{1} & {\text{x}}_{2} & 0_{2} & \text{C} \end{array} \end{array}$$

E = Experimental, C = control

x_1_ = treatment with flipped classroom

x_2_ = treatment without flipped classroom

Y = moderator variable (attitude)

O_1_, O_1_ = Pretest measurement

0_2_, 0_2_ = Post-test measurement

**Figure 1:**
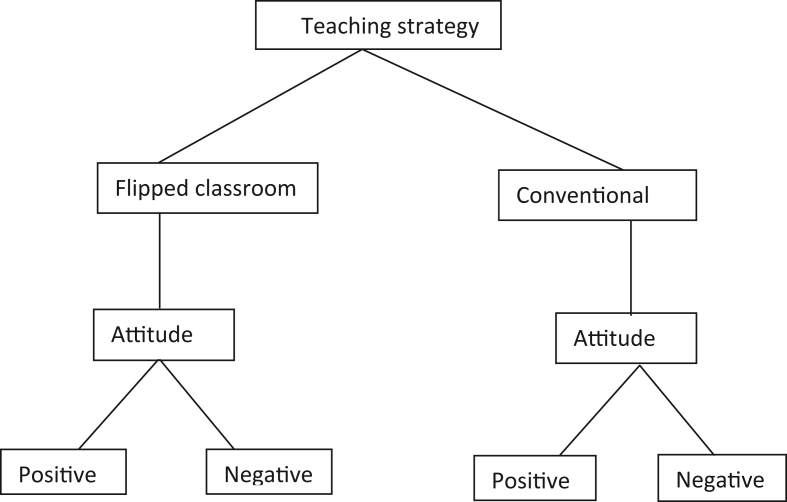
Features of 2 × 2 factorial design.

The design shows that:

i) Treatment was administered to both experimental and control groups (2 levels)
ii) Attitude was categorized as positive and negative (2 levels). A score of 30–89 in the attitude questionnaire was classified as negative and 90 to 120 was positive. This categorization was used for both flipped and conventional classroom. Figure 1 shows the pictorial representation of the design and Table 1 shows the design of the study.

**Table 1:** Design of the study.

Group	I	Pre-test/pre attitude	Treatment (6week)	Post-test/post attitude
Experimental		Pre performance test/pre attitude	XFC	Postperformance test/Attitude test in Flipped classroom
Control	I	preperformance test/pre attitude	XCL	post performance test/Attitude test

Note. I = intact class; XFC = Flipped Classroom; XCL = Conventional Learning.

In this study, factorial design was adopted because it allowed the evaluation of the outcome of the independent variables separately and their joint effects (Onwiduokit, 2000). Teaching in the experimental class was done using the flipped strategy while non flipped strategy was used for the control group. The experimental and control group were given pre-test and post-test. The pre attitude and post attitude test were also administered to the experimental group. The sample was made up of (100) BSc (Ed) chemistry undergraduate students. A break-down of this figure showed 60 undergraduate students in experimental and 40 undergraduates in the control groups.

Instrumentation Three instruments were used for this study. They were: videos of redox reactions, Redox reaction test (Redox-Test) and chemistry Attitude questionnaire (CAQ). Redox-Test was an instrument developed by the researcher for data collection. It was a 20 item five response option objective test. This served as the pretest and posttest after validation. Redox-Test items was rearranged with its options in pretest and posttest having variant numbering to give an obscure idea that the test was not the same. Redox-Test items were selected to include all the lessons in Redox reactions (Appendix A).

Redox-Test was face and content validated before reliability was carried out. Reliability denotes the measure of accuracy with which a test measures what it was planned to measure (Onwioduokit, 2005). The reliability test of the questionnaire was carried out with thirty second year BSc (Ed) chemistry students of Cross River State University. These students in this University are similar to those in University of Calabar that were used for the research. The essence of this test was to ascertain the reliability of the instrument. The result obtained in this administration was subjected to Kuder Richardson's formular-20 analysis. Reliability coefficient of the test instrument was 0.81. A reliability coefficient of 0.50 and above is good and high enough to justify the usage of an instrument (Joshua, 2005). Therefore, the estimates obtained were high enough to justify the use of the instruments in conducting the research.

The chemistry Attitude Questionnaire (CAQ) questionnaire (Appendix B). CAQ was formed, validated by Salta and Tzougraki (2004). This was to access chemistry students' attitudes. The CAQ questionnaire was made up of 30 items that had four concepts on a 4-point Likert scale. Response options ranged from ‘‘strongly disagree’’ to ‘‘strongly agree’’. Strongly disagree’’ scored 1, disagree 2, agree 3 and strongly agree 4 points respectively. The overall attitude score that respondents will have to fall in between 30–120. This scoring makes the respondents with the minimum score to be 30 and 120 as the maximum score. The 4 concepts used in the questionnaire that had 30 items are shown in Table 2. As represented in Table 2, CAQ estimated the attitude of student towards chemistry by level of relevance, simplicity of the concept, motivation and benefits of chemistry in their imminent occupation/profession. The reliability test of the questionnaire was carried out with thirty second year BSc (Ed) chemistry students of Cross River State University. These students in this University are similar to those in University of Calabar that were used for the research. The essence of this test was to establish the reliability of the instrument. The Cronbach's alpha for the 4 concepts of CAQ during the trial test was 0.85 which is appropriate because of the size of the sample that was not large. Few changes on the CAQ questionnaire were made, like ‘‘course’’ was reverted to ‘‘concept’’ to agree with the context of participating students. CAQ was adapted and adopted in the main study. This instrument was apportioned before and after teaching using the flipped classroom.

**Table 2:** Descriptive statistics for pre and post attitude.

	Mean	N	Std. Deviation	Std.Error
Pre attitude	40.88	60	8.675	1.120
Post attitude	76.73	60	14.630	1.889

Sampling technique 100 second year Bachelor of Chemistry Education students of University of Calabar in 2019/2020 academic session formed the population of this study. Sixty (n = 60) students were selected in a random form from the population and assigned to experimental and forty (n = 40) students were assigned to the control. Both the experimental and control groups participated in the pretest/posttest and pre attitude/post attitude test.

### 2.2 Procedure for data collection

This research was carried out during the first semester of 2019/2020 academic year for the experimental and control. This covered 6 weeks (one month two weeks) for the control and experimental group. The choice of 6 weeks was to avert maturation, which can compromise the internal validity.

Each week comprised 2 h of teaching using the flipped classroom for the experimental group and the conventional method for the control group. Before treatment, the subjects both in experimental group and control were given a pretest to obtain a base for comparability. The test scores on the pretest described the cognitive entry knowledge possessed by the students concerning the selected dependent variable. Pre attitude test was also given to the experimental group and control to ascertain their attitude toward chemistry learning before instruction with the flipped classroom and conventional method.

In the experimental group, videos of what was to be taught to subjects were sent to their WhatsApp group platform (Appendix C). This was done after the pretest and pre-attitude were administered. This was done a week before instruction by the researcher. One week was given to students to watch the videos and prepare their questions for the class interaction. The subjects in experimental groups were grouped into sub-groups of ten for instructions. This was done by the researcher's assistant and subjects turning on the required video in the classroom to refresh their minds. The researcher assistants then asked students to discuss the video they have watched. Students had written questions at home on what does not seem clear and brought to the classroom. All such questions were answered by the research assistants in the classroom. In the control group, subjects were taught immediately the pretest and pre attitude was administered conventionally.

The experimental group was treated with the flipped classroom while the control groups were not given treatment but taught their normal science curriculum. Post-test was re-administered after the end of teaching to the control and experimental groups. The instruments served both as the pretest and the posttest. Certain minor changes in numbering on the test instrument were done to give a vague impression that the post-test was essentially different from the pre-test taken previously to guide against testing effect. Post attitude test was administered to the experimental group and control after instructions. The effect of non-equivalence between the experimental and control groups was minimized by the use of analysis of covariance as a statistical tool. It allows for statistical rather than experimental control of variables (Onwiduokit, 2000).

Concerning the internal validity of the experiments, efforts were made to remove or minimize the potential threats. Denga and Ali (1998) have enumerated threats to the internal validity of a study. These are maturation, selection, history, testing, instrumentation, statistical regression, and mortality. Maturation was taken care of as the study did not last for a very long time and both the experimental and control had the study at the same time. Random sampling took care of selection, while a change of numbers in pretest-posttest and pre attitude and post attitude took care of testing. The other factors were annulled through the use of analysis of covariance (ANCOVA).

The Chemistry achievement test (Cat) contained twenty multiple choice objective test items with a five response option format. Each item had four distractions and one correct option. Every correct option in each instrument scored 1 mark and wrong option scored zero mark. The maximum marks for all 20 items in each instrument were 20 marks.

### 2.3 Data analysis

CAQ data obtained from the attitude questionnaire was analyzed using inferential and descriptive statistics. This was used in computing and comparing the mean achievement scores for pre/post-CAQ. t-test for correlated data was applied to ascertained if the derived mean is significant or not. CAT data obtained was analyzed using descriptive and inferential statistics in computing and comparing mean achievement scores for pre/post-test. Analysis of covariance (ANCOVA) was used for data analysis.

Students were required to tick to the under listed phrases on a Likert four-point scale. Concept Questionnaire items Example of items as represented on Table 2. The relevance of Chemistry concept 5, 12, 13, 15 and 20 Item 12: Advancement of chemistry enhances the quality of our lives. Simplicity of chemistry concept 2, 7, 17, 18, 24 and 26 Item 18: I find the use of chemical symbols easy. Motivation in the chemistry concepts 1, 3, 9, 10, 16, 19, 21, 23 and 25 Item 25: I find chemistry concepts very interesting. Utilization of chemistry for imminent profession 14, 22 and 30 Item 14: My imminent profession is independent of chemistry content knowledge. Data collected from the pre-and post-attitude tests were analyzed using descriptive statistics and parametric testing after the standard-issue of the data was ascertained.

Table 2 showed the mean post-test attitude score (M = 76.73, S.D. = 14.630) was higher than the pre-test score (M = 40.88, S.D. = 8.67). A quick look at the questionnaire items in their distinct concepts showed that, post-mean differences for separate concepts presented in the CAQ questionnaire were different.

Table 3 indicated that the mean attitude scores difference between the pre attitude and post attitude for simplicity and motivation concepts in the CAQ questionnaire was high. As shown in Table 3, the post attitude score for simplicity was 26.25 and the pre attitude score was 9.97 the difference in the mean score is 16.28. The motivation post means the score was 30.29 and the mean pre score was 12.37, the difference is 17.92. The result indicated a positive attitude in students' perception of simple and motivational concepts in chemistry. Utilization had a post attitude mean of 6.85 and a pre attitude means a score of 5.77. The difference between the pre and post-attitude mean score is 1.08. Even though students' response to the concept of relevance in the questionnaire at the pretest stage was high mean = 12.77 the mean score increase of the post attitude was small (13.34). There was a small increase in the mean score of the concept under investigation of 0.57.

**Table 3:** Descriptive statistics for pre and post-attitude Attitudes.

Attitudes				Mean	N	Std deviation	Std error
Group	1	Simplicity	pre	9.97	60	3.344	.432
		Simplicity	post	26.25	60	4.011	.353
Group	2	Motivation	pre	12.37	60	3.664	.473
		Motivation	post	30.29	60	6.068	.783
Group	3	Utilization	pre	5.77	60	1.329	.172
		Utilization	post	6.85	60	3.177	.410
Group	4	Relevance	pre	12.77	60	3.824	.494
		Relevance	post	13.34	60	3.781	.488

Before flipped classroom teaching, students viewed chemistry as a hard subject and therefore had a low mean score of 9.97 for the simplicity concept of the questionnaire. After being taught using the flipped classroom their perception dropped and their mean score of 9.97 increased to 26.25. Table 3 above showed that a large variation in the pre and post-attitude test scores was largely accounted for by 2 concepts, which were ''simplicity of the subject'' (M = 9.97, 26.2) and ''motivation in the subject'' (M = 12.37, 30.29). This indicated that before teaching using flipped classroom strategy students presumed the concepts to be difficult and did not exhibit much interest in them. A paired sample t-test for pre and post-test scores was conducted after it was ascertained that the data had a normal distribution. The results are shown in Table 4.

**Table 4:** Paired Samples Test for pre and post-attitude.

	Mean	Paired Differences	Std. Error	95%Confidence Interval of the Difference				
		Std. Deviation	Mean	lower	Upper	t	df	Sig (2- tailed
Pre-attitude, post -attitude	35.85	12.63	1.63	-39.11	-32.59	21.99	59	0.000

Table 4, showed a statistically significant higher post-test mean than the pre-test mean, with a significantly higher positive attitudes towards chemistry, t (59) = 21.99, p < 0.05 at 95% confidence interval. The results also revealed that BSc.(Ed) 2 nd year chemistry students displayed an improvement in their attitude towards chemistry when taught using flipped classroom strategy.

Table 5 was used to find out if the difference in the pre/post attitude in the CAQ concepts were statistically significant. That Table 5 showed that the concepts ‘’Motivation’’ and simplicity’’ concepts showed a significant difference in the pre/post attitude test. This implied that the used of flipped classroom strategy positively affected their perception of chemistry concepts in terms of simplicity of questions and also their motivation to study chemistry. Significant differences in the attitudes towards CAQ concept in simplicity of chemistry as a subject and motivation toward the study of chemistry indicated that academic performance in chemistry can be influenced by the used of the flipped classroom. The change in attitudes of utilization and relevance were not significant. This can be seen as indicated in Table 5 that t (59) = 1.324, p > 0. 191 for relevance and t (59) = .506, p > 0. 614 at 95% confidence interval. The explanation of this result could be that teaching with the flipped classroom did not change the relevance of chemistry and students utilization of chemistry as a subject as they may be aware of these before the flipped classroom strategy. Teaching using the flipped classroom did not affect students' attitude in terms of utilization and relevance.

**Table 5:** Paired samples t test of CAQ concepts.

	Mean	Std. Deviation	Std. Error Mean	95% Confidence Interval of the Difference Lower Upper		t	df	Sig. (2-tailed
Pre-simplicity, Post -simplicity	7.27	4.50	.58	6.10	8.43	12.50	59	000
Pre-motivation, post- motivation	5.917	3.19	.41	-6.74	-5.09	14.35	59	000
Pre-utilization, post-utilization	.57	3.32	.43	-1.42	.29	1.32	59	.191
Pre-relevance, Post- relevance	.38	5.86	.76	-1.13	1.90	.506	59	.614

The next research phase sought to find out the academic achievement of second year undergraduate chemistry education students taught with and without the flipped classroom strategy. The result was presented on Tables 6 and 7.

**Table 6:** Mean standard deviation of influence of treatment and attitude on the academic performance of undergraduate chemistry students.

Treatment	Attitude	Mean	Std. Deviation	N
Experimental	Negative	12.96	2.549	27
	Positive	14.52	2.526	33
	Total	13.82	2.633	60
Control	Negative	10.25	1.849	32
	Positive	13.75	1.282	8
	Total	10.95	2.241	40
Total	Negative	11.49	2.569	59
	Positive	14.37	2.343	41
	Total	12.67	2.846	100

**Table 7:** Summary of 2 × 2 analysis of covariance of influence of treatment and attitude on the academic performance of undergraduate chemistry students.

Sources of variation	Type III Sum of Squares	df	Mean Square	F	Sig.	Partial Eta Squared
Corrected Model	329.078a	4	82.270	16.522	.000	.410
Intercept	2766.319	1	2766.319	555.566	.000	.854
Pre test	17.674	1	17.674	3.549	.063	.036
Treatment	46.781	1	46.781	9.395	.003	.090
Attitudes	119.866	1	119.866	24.073	.000	.202
Treatment Attitude	24.440	1	24.440	4.908	.029	.049
Error	473.032	95	4.979			
Total	16855.000	100				

R Squared = .410 (Adjusted R Squared = .385).

The result of the analysis displayed in Table 6 showed that students taught Redox reactions using flipped classroom had higher mean achievement (M = 13.82) in comparison to those taught without the flipped classroom strategy which had a mean of M = 10.95. That same Table 6 also showed that the mean score of students taught using the flipped classroom with a positive attitude toward chemistry was higher than those with a negative attitude toward chemistry 14.52 and 12.96 respectively.

As shown in Table 7, treatment∗attitude is the interaction of teaching strategies (flipped and conventional) and attitudes on chemistry students' academic achievement. This was used to find out how attitude influence the academic achievement of chemistry students when taught with or without the flipped classroom. The result of the analysis as displayed in Table 7 indicated a high significant F (1, 95) value of 9.395, p=<. 05 for treatment (use of the flipped and conventional classroom) and F (1,95) = 24.073, p=<. 05 for attitude was significant and the interaction between treatment and attitude was significant as F1, 95 = 4.908, p=<. 05. This results implies that students’ attitude influence their academic achievement when taught with and without the flipped classroom. The influence of attitude on the academic achievement of chemistry students when taught using the flipped and conventional strategy as indicated in Table 7 contributed 4.9% (.049) of the total variance in academic achievement. This answers the question that sought to find out if attitude of students when taught with and without the flipped classroom influenced their academic achievement in chemistry.

Treatment (flipped classroom) contributed 9% (.090) of the total variance in academic achievement. Since the p-value (.003) for treatment was lower than 0.05, the null hypothesis speculated that there is no significant difference between the posttest performance of second-year undergraduate taught Redox reactions using the flipped classroom and those taught without the strategy was rejected and the alternate hypothesis accepted.

The second research question sought to find out if there is a significant influence of attitudes on chemistry students' performance in chemistry? The mean of students with a positive attitude toward chemistry was higher than those with a negative attitude toward chemistry 14.37 and 11.49 respectively. Attitude was significant as F (1,95) = 24.073, p=<. 05. The p-value of .000 was lower than the 0.05 significance level and therefore the result is significant. This answered the second research question that there exists a significant influence of attitudes on chemistry students' performance in chemistry. The result in Table 7 also showed that there was a significant interaction effect of treatment and attitude on the academic performance of chemistry students (F1, 95 = 4.908, p=<. 05).

## 3 Discussion

The result of this study showed that second year B.Sc (Ed) chemistry students' attitudes towards chemistry were positively affected by the flipped classroom strategy. The results of this research indicated that positive attitude had positive impact on the academic performance of B.Sc(Ed) chemistry students. It also showed that the flipped classroom strategy improved students' academic performance in Redox reaction test in chemistry. Some factors like the availability of power, network strength and student–facilitator interactions that may have influenced students' attitudes and academic performance were not investigated in this study. Flash back on literature shows that the findings collaborated other studies that investigated the effects of flipped classroom strategy on academic performance of students (Kim, Jung, de Siqueira, and Huber, 2016; Bhagat, Chang, and Chang, 2016; Gopalan, 2018. Elian and Hamaidi, 2018; Covill and Cook, 2019; Busebaia and John, 2020). In these studies flipped classroom strategy were reported to have increased student academic performance in chemistry. On the influence of flipped classroom on students' attitude, the results were in consonance with previous study by (Hunley, 2016; Sterner et al., 2017; Karadag and Keskin, 2017) had positive impact on students’ attitudes learning in general.

The results also showed that positive attitude of students' brought about an increase in academic performance in the Redox reaction test in chemistry. Students with positive attitude achieved more than students with negative attitude may be because flipped classroom used videos as mode of instruction. Students' attitude goes a long way to determine what a student can achieve. Whatever a student has consciously set out his/her mind to do that can be done. A positive attitude is like a mirror that reflects what the student is about to do and therefore energizes the student to accomplish it. A negative attitude dampens one's morale which weakens one and thereby leading to low achievement. The emotions and mental entities in the flipped classroom could have propelled students to learn better since they enjoy watching videos. A recapture of the literature showed that this study agreed with other studies that sought the effects of students attitude on their academic performance (Paramitha (2017); Sterner et al., 2017; Karadag and Keskin (2017); Hunley, 2016; Marcela and Mala, 2016; Hacieminoglu (2015); Juma and Ahmad, 2012).

Flipped classroom impacted positively on students' attitude may be related also to the fact that students enjoy playing with their phones and laptop. Playing of video was like fun and therefore they had positive attitude in the flipped classroom. The interactive classes with their smart phones make them happy and learning less stressful. Saglam and Arslan (2018), reported that when the flipped classroom strategy was used in teaching in higher school, students’ academic performance was enhanced and they developed positive attitude toward English language.

In line with these findings, Marcela and Mala, 2016 study indicated that a positive attitude gives rise to positive results while a negative attitude turns out a negative result. Hacieminoglu (2015) research on Students' Attitudes toward Science, showed that students who had a more positive attitude towards science had higher achievement scores. In the same vein, Juma and Ahmad, 2012 reported an expressive affinity between learners' attitudes concerning Information Technology and scholars' intellectual achievement. It indicated that intellectual achievement and attitudes are interwoven. Paramitha's (2017), study pointed out the fact that students' interest and attitude in learning are the predominant factors in the mastery of any subject matter. This research indicated the importance of positive attitudes in increasing student's academic achievement.

Flipped classroom strategy improved students’ academic performance. This could have been because; students in the flipped classroom had reviewed learning materials at home and were ready to harness what was previously learned at home. The teacher commenced the teaching process by checking students' degree of comprehension and reflected on what was previously studied at home. Students were at liberty to play the educational video for mastery of concept taught as against the conventional method. Flipped classroom was an active classroom setting thereby making learning more rewarding.

This study was in collaboration the study of Flynn (2018) whose study investigated the effect of flipped classroom on undergraduate students of University of Ottawa. Courses discussed were organic chemistry and spectroscopy. The result indicated that the flipped courses had higher mean score and students' satisfaction compared to courses taught in an active lecture format. Flipped classroom also improved student performance compared with that of lecture-based traditional teaching practice in a study carried out in Introductory Physiology course by Gopalan (2018). In the same vein, Karadag and Keskin (2017) study on the flipped classroom and attitude of mathematics students agreed with this study as they reported high level of student's achievement in a flipped classroom. Sirakaya and Ozdemir (2018), study on the flipped classroom indicated a meaningful disparity between groups with regards to academic attainment and motivation. The findings of a study done by Busebaia and John (2020) on undergraduate pediatric nursing students supported the use of flipped classroom in learning pediatric course content.

Contrary to these findings, Covill and Cook (2019), observed that learning outcomes of traditional lecture and flipped method classes were similar in their study. Another study by Cabi (2018) reported a meaningful effect of a flipped environment on improving scholars’ academic attainment.

### 3.1 Limitations

In this study intact classes were used and the researcher could not select students into the experimental and control groups. They were merely assigned into such groups. Significant differences in the academic performance and student attitude towards chemistry indicated that other variables which were not sought for, may have impacted on the positive change in the academic performance and attitude change. The particular chemistry topic selected was not the only difficult topic in chemistry according to examiners report. However, the primary aim of the application of the flipped strategy was to use what instructional approach students like to be taught with, that being videos. Other factors which include the method, class size and interpersonal relationship may have impacted on the negative attitude of students before the use of the flipped classroom.

## 4 Conclusion

The inferences, which was drawn from the findings of this research are connected to the positive effect of the flipped classroom strategy on students’ attitudes towards chemistry and academic performance in chemistry. Flip classroom strategy provided a platform for the students to learn at their own space and repeatedly to gain understanding of abstract chemistry concepts. Flipped classroom assisted students to learn at their pace according to their individual differences which led to better understanding of abstract chemistry concepts. Even though flipped classroom requires the use of data to download videos, students can transfer videos to each other.

### 4.1 Implication for further research

The following implications for further research might be suggested given the results of the study: In chemistry learning, the flipped classroom could be used to improve students' academic performance in chemistry and enhance positive attitudes towards chemistry. It has also shown the importance of communication technology when used in the classroom setting as well as the student-centered type of learning. Out of the four components of attitude studied, simplicity items and motivation items showed dramatic improvement when flipped classroom was used as a method of instruction.

Workshops may be organized to train teachers on how to implement flipped classrooms in their teaching and learning process. There should be a resource base where videos and hand out for teaching chemistry be kept for all chemistry teachers to work cooperatively. Teachers' training programmes should provide the student teachers with activities to get them to participate in flipped classroom practices. This will equip incoming teachers with the expertise to implement flipped classrooms in their teaching.

A study can be conducted to investigate the effect of the flipped classroom on other psychological concepts like self-esteem, locus of control, and interpersonal intelligence of chemistry students when a flipped classroom is used during teaching. Research may also be carried out on teachers' variables and their use of flipped classroom strategies. Such variables can include; age, gender, years of teaching, and academic qualification.

## Declarations

### Author contribution statement

CECILIA OBI NJA: Conceived and designed the experiments; Analyzed and interpreted the data; Wrote the paper.

RICHARD EKONESI ORIM: Analyzed and interpreted the data.

HOPE AMBA NEJI: Performed the experiments.

JOHN OKPA Ukwetang: Analyzed and interpreted the data.

UDUAK EDET UWE: Analysis tools or data.

MARY ANARI IDEBA: Analysis tools or data; Wrote the paper.

### Funding statement

This research did not receive any specific grant from funding agencies in the public, commercial, or not-for-profit sectors.

### Data availability statement

Data will be made available on request.

### Declaration of interests statement

The authors declare no conflict of interest.

### Additional information

No additional information is available for this paper.

## Appendix A

### Appendix A. Redox test item (pretest).

Attempt all questions. Tick only one option that is correct to you.

1 When sodium atom forms an ion Na+
  a) It gains one electron.
  b) It gains one proton.
  c) It achieves a noble gas configuration.
  d) Its atomic number increases.
  e) Its shares electron
2 Which specie undergo reduction in the reaction represented by the equation below
H2S(aq) + FeCl3(aq) ----→ S(s) + 2HCl + 2FeCl2(aq)
  a) Fe3+
  b) H2S
  c) Cl
  d) S
  e) H
3 What is the oxidation number of chromium in K2CrO4?
  a) +1
  b) +2
  c) +4
  d) +6
  e) 5
4 An atom of an element X gains two electrons. The symbol of the ion formed is
  a) X+
  b) X2+
  c) X2-
  d) X
  e) 2X
5 Consider the redox reaction as represented by the following equation
I2 (aq) + 2S2O3 ^2-^ (aq) -----> 2I--(aq) +S4O6 ^2-^(aq) which of the species in the equation is reduced?
  a) S4O6^2-^ (aq)
  b) S2O3^2--^ (aq)
  c) I^2-^ (aq)
  d) I^−-^ (aq)
  e) O3^2-^(aq)
6 Reduction is the process of
  a) Loss of electron
  b) Loss of hydrogen
  c) Loss of oxygen
  d) Addition of electromagnetic elements
  e) increase in oxidation number
7 In the reaction CH4 + 2O2 -----> CO2 + 2H2O, what is the oxidation number of carbon in CH4
  a) +4
  b) -4
  c) +2
  d) -2
  e) +1
8 In the reaction in question 7 above 2O2 is
  a) Oxidized
  b) Reduced
  c) Neutralized
  d) Promoted
  e) Magnetized
9 In the half reaction below Na -----> Na+ + e-. Na has undergone
  a) Oxidation
  b) Reduction
  c) Neutralization
  d) Precipitation
  e) sublimation
10 In the redox reaction below
Al + Ni^+2^ ----> Al^+3^ + Ni is balanced
  a) By making sure the number of atoms and charges are the same on both sides
  b) Only charges are the same on both sides
  c) Only atoms are the same on both sides
  d) Not checking check for atoms and charges on both sides
  e) Adding water at the right hand side of the equation.
11 In the redox reaction below, how many electrons are added to balance the equation?
Zn + BrO3 → Zn^+2^ + Br
  a) 6
  b) 4
  c) 5
  d) 3
  e) 2
12 During balancing of redox reaction in acidic medium, --------- is added
  a) Hydrogen ion and water
  b) Hydrogen ion and hydroxyl ion
  c) Water and oxygen
  d) Water and hydroxyl ion
  e) Oxygen and hydrogen
13 In the redox reaction
Zn + BrO3 ----> Zn+2 + Br-
The number of hydrogen ion required to balance the equation is
  a) 6
  b) 5
  c) 4
  d) 3
  e) 2
14 During balancing of redox reaction in alkaline medium ------ is added
  a) Water and hydroxyl ion
  b) Water and hydrogen ion
  c) Hydroxyl ion and hydrogen ion
  d) Hydroxyl ion and oxygen
  e) Hydroxyl ion and base
15 The total number of charges in the redox reaction below when balanced is
Al + ClO4^-^ ----> Al (OH)4 ^-^ + Cl
  a) 12
  b) 11
  c) 24
  d) 8
  e) 6
16 Oxidizing agent ------
  a) Gives out electrons
  b) Gain electrons
  c) Share electrons
  d) Are inert
  e) Join electron
Use this redox equation to answer question 17-20
MnO4^-^(aq) +8H^+^ + 5Fe^2+^(aq) ----->5Fe^3+^ + Mn^2+^(aq) + 4H20 (l)
17 Manganese has undergone
  a) Oxidation
  b) Reduction
  c) Precipitation
  d) Neutralization
  e) Elimination
18 Oxidation state of manganese has changed from
  a) +7 to +5
  b) +8 to +6
  c) +7 to +4
  d) +8 to +4
  e) +8 to +3
19 Iron has undergone
  a) Oxidation
  b) Reduction
  c) Precipitation
  d) Neutralization
  e) Polarization
20. Oxidation state of Iron has changed from
  a) +6 to +7
  b) +2 to +3
  c) +2 to +1
  d) +3 to +4
  e) +4 to +5

### Appendix A. Redoxt test item (post test).

Attempt all questions. Tick only one option that is correct to you.

Use this redox equation to answer question 1-4.

MnO4^-^ (aq) +8H+ + 5Fe^2+^(aq) ----->5Fe^3+^ + Mn^2+^(aq) + 4H20 (l)

1. Manganese has undergone
  a) Oxidation
  b) Reduction
  c) Precipitation
  d) Neutralization
  e) Elimination
2. Oxidation state of manganese has changed from
  a) +7 to +5
  b) +8 to +6
  c) +7 to +4
  d) +8 to +4
  e) +8 to +3
3. Iron has undergone
  a) Oxidation
  b) Reduction
  c) Precipitation
  d) Neutralization
  e) Polarization
4. Oxidation state of Iron has changed from
  a) +6 to +7
  b) +2 to +3
  c) +2 to +1
  d) +3 to +4
  e) +4 to +5
5 When sodium atom forms an ion Na+
  a) It gains one electron.
  b) It gains one proton.
  c) It achieves a noble gas configuration.
  d) Its atomic number increases.
  e) Its shares electron
6. Which specie undergo reduction in the reaction represented by the equation below
H2S(aq) + FeCl3(aq) ----→ S(s) + 2HCl + 2FeCl2(aq)
  a) Fe3+
  b) H2S
  c) Cl
  d) S
  e) H
7 What is the oxidation number of chromium in K2CrO4?
  a) +1
  b) +2
  c) +4
  d) +6
  e) 5
8 Reduction is the process of
  a) Loss of electron
  b) Loss of hydrogen
  c) Loss of oxygen
  d) Addition of electromagnetic elements
  e) increase in oxidation number
9 In the reaction CH4 + 2O2--- > CO2 + 2H2O, what is the oxidation number of carbon in CH4
  a) +4
  b) -4
  c) +2
  d) -2
  e) +1
10 In the reaction in question 9 above 2O2 is
  a) Oxidized
  b) Reduced
  c) Neutralized
  d) Promoted
  e) Magnetized
11 In the half reaction below Na -----> Na+ + e-. Na has undergone
  a) Oxidation
  b) Reduction
  c) Neutralization
  d) Precipitation
  e) sublimation
12 In the redox reaction below
Al + Ni+2 ----> Al+3 + Ni is balanced
  a) By making sure the number of atoms and charges are the same on both sides
  b) Only charges are the same on both sides
  c) Only atoms are the same on both sides
  d) Not checking for atoms and charges on both sides
  e) Adding water at the right hand side of the equation.
13 In the redox reaction below, how many electrons are added to balance the equation?
Zn + BrO3 → Zn+2 + Br
  a) 6
  b) 4
  c) 5
  d) 3
  e) 2
14. In this redox reaction
Zn + BrO3 ----> Zn^+2^ + Br^-^
The number of hydrogen ion required to balance the equation is
  a) 6
  b) 5
  c) 4
  d) 3
  e) 2
15 During balancing of redox reaction in alkaline medium ------ is added
  a) Water and hydroxyl ion
  b) Water and hydrogen ion
  c) Hydroxyl ion and hydrogen ion
  d) Hydroxyl ion and oxygen
  e) Hydrogen ion and base
16 During balancing of redox reaction in acidic medium, --------- is added
  a) Hydrogen ion and water
  b) Hydrogen ion and hydroxyl ion
  c) Water and oxygen
  d) Water and hydroxyl ion
  e) Oxygen and hydrogen
17. The total number of charges in the redox reaction below when balanced is
Al + ClO4^-^ ----> Al (OH)4 ^-^ + Cl
  a) 12
  b) 11
  c) 24
  d) 8
  e) 6
18. Oxidizing agent ------
  a) Gives out electrons
  b) Gain electrons
  c) Share electrons
  d) Are inert
  e) Join electron
19 An atom of an element X gains two electrons. The symbol of the ion formed is
  a) X+
  b) X2+
  c) X2-
  d) X
  e) 2X
20. Consider the redox reaction as represented by the following equation
I2 (aq) + 2S2O3^2-^(aq) -----> 2I^−-^ (aq) +S4O6 ^2-^(aq) which of the species in the equation is reduced?
  a) S4O6^2-^(aq)
  b) S2O3^2--^(aq)
  c) I2^-^ (aq)
  d) I^−-^(aq)
  e) O3^2-^ (aq)

### Appendix B

CHEMISTRY ATTITUDE QUESTIONNAIRE (CAQ)

Respond to the following statements. SA A D SD.

1. I like chemistry course more than the others.
2. Chemical images do not resemble Chinese to me.
3. I might want to have chemistry exercises all the more frequently.
4. The advancement of chemistry is not answerable for some environmental problems.
5. Chemistry information is valuable to decipher numerous parts of our regular day to day existence.
6. Chemistry course is identified with different courses.
7. I address chemistry practices without any problem.
8. Chemistry course helps the improvement of my reasonable abilities.
9. During chemistry exercises, I am not exhausted.
10. Chemistry information will not be futile after my graduation.
11. Chemistry information is fundamental for understanding different courses.
12. The advancement of chemistry improves the nature of our lives.
13. Chemistry is our expectation for tackling numerous environmental problems.
14. My future profession is not autonomous of chemistry information.
15. The advancement of chemistry adds to the improvement of a country.
16. Chemistry is an exceptionally sophisticated subject for our necessary education.
17. I put forth numerous attempts to get chemistry.
18. I discover the utilization of chemical images simple like stroll over.
19. The profession of a scientist is not one of the less alluring.
20. Each resident should have chemistry information.
21. I do not disdain chemistry courses.
22. Chemistry information is important for my future profession.
23. I might want to have more chemistry exercises.
24. I comprehend the chemistry concepts without any problem.
25. I discover the chemistry course fascinating.
26. At the point when I attempt to address chemistry works out, my psyche goes clear.
27. Individuals are concerned with chemistry applications.
28. The advancement of chemistry does not demolish the states of living.
29. I'm equipped for deciphering my general surroundings utilizing chemistry information.
30. I might want to turn into a chemist when I finish school.

### Appendix C

(see supplementary file for the videos).
